# Preservation of nautilid soft parts inside and outside the conch interpreted as central nervous system, eyes, and renal concrements from the Lebanese Cenomanian

**DOI:** 10.1186/s13358-021-00229-9

**Published:** 2021-07-23

**Authors:** Christian Klug, Alexander Pohle, Rosemarie Roth, René Hoffmann, Ryoji Wani, Amane Tajika

**Affiliations:** 1 Paläontologisches Institut Und Museum, Universität Zürich, Karl-Schmid-Strasse 4, 8006 Zürich, Switzerland; 2 Institute of Geology, Ruhr-Universität Bochum, Mineralogy, & Geophysics, 44801 Bochum, Germany; 3 Faculty of Environment & Information Sciences, Yokohama National University, Yokohama, 240-8501 Japan; 4 Division of Paleontology (Invertebrates), American Museum of Natural History, Central Park West 79th Street, New York, NY 10024 USA; 5 University Museum, University of Tokyo, 7-3-1 Hongo, Bunkyo-ku, Tokyo, 113-0033 Japan

**Keywords:** Cephalopoda, Nautilida, Cretaceous, Anatomy, Konservat-Lagerstätte, Taphonomy

## Abstract

Nautilid, coleoid and ammonite cephalopods preserving jaws and soft tissue remains are moderately common in the extremely fossiliferous Konservat-Lagerstätte of the Hadjoula, Haqel and Sahel Aalma region, Lebanon. We assume that hundreds of cephalopod fossils from this region with soft-tissues lie in collections worldwide. Here, we describe two specimens of *Syrionautilus libanoticus* (Cymatoceratidae, Nautilida, Cephalopoda) from the Cenomanian of Hadjoula. Both specimens preserve soft parts, but only one shows an imprint of the conch. The specimen without conch displays a lot of anatomical detail. We homologise the fossilised structures as remains of the digestive tract, the central nervous system, the eyes, and the mantle. Small phosphatic structures in the middle of the body chamber of the specimen with conch are tentatively interpreted as renal concrements (uroliths). The absence of any trace of arms and the hood of the specimen lacking its conch is tentatively interpreted as an indication that this is another leftover fall (pabulite), where a predator lost parts of its prey. Other interpretations such as incomplete scavenging are also conceivable.

## Introduction

As shown by Clements et al. (2016), the preservation of soft-tissues of cephalopods is linked with their physiology to some degree. Additionally, an outer shell as for example in ammonoids and nautiloids also hampers the study of soft tissues, because even if they are preserved, they are either covered by shell remains (or their replacement) or surrounded by cements or sediment. Hence, the greatest likelihood to discover soft parts of ammonoids or nautilids is given in such Konservat-Lagerstätten (= conservation deposits; Seilacher, 1970), where soft parts became quickly phosphatised or pyritised and aragonite shells were dissolved. There are records of cephalopod soft parts and there is some potential for such preservation in, e.g., the Ordovician Soom Shale in South Africa (Gabbott, 1999), the Devonian Hunsrück Slate in Germany (De Baets et al., 2013; Stilkerich et al., 2017), the Devonian Hangenberg Black Shale in Morocco (Klug & Vallon, 2019; Klug et al., 2016a), the Carboniferous of Bear Gulch (Doguzhaeva et al., 2007; Klug et al., 2019; Landman et al., 2010; Mapes et al., 2019), the Carboniferous of the Itararé‑Formation in Uruguay (Closs, 1967; Lehmann et al., 2015), the Triassic Thaynes Group in the USA (Doguzhaeva et al., 2018), the Jurassic of Christian Malford and Dorset (Hart et al., 2020; Wilby et al., 2004, 2008), the Jurassic of Eichstätt, Nusplingen, Painten and Solnhofen (e.g., Dietl & Schweigert, 2011; Fuchs, 2006a; Klug et al., 2015, 2016b, 2021a, 2021b; Schweigert, 2009, 2021), the Cretaceous of Germany (Klug & Lehmann, 2015; Klug et al., 2012), and, last but not least, the Cretaceous of Lebanon (Engeser & Reitner, 1986; Fuchs & Larson, 2011a, 2011b; Fuchs, 2006a, 2006b; Jattiot et al., 2015; Klug et al., 2020, 2021a; Lukeneder & Harzhauser, 2004; Roger, 1946; Wippich & Lehmann, 2004; Woodward, 1883, 1896).

Here, we describe remains of nautilids from Hadjoula (also transcribed as Hjoula or Hjula) in Lebanon. There are various localities, where, like in Hadjoula, fossils are quarried commercially from the Cenomanian platy limestones of the Aitou Formation (Ferry et al., 2007). Commercial quarries also exist at Sahel Aalma and Haqel (also transcribed as Haqil). Already in the nineteenth century, this region northeast of Beirut became famous for its very rich early Cenomanian (Keupp et al., 2016; Wippich & Lehmann, 2004) occurrences of articulated fossils of highly diverse fish (actinopterygian, sarcopterygian, chondrichthyan), echinoderms, arthropods and cephalopods (e.g., Foord & Crick, 1890; Lewis, 1878; Naef, 1922; Woodward, 1883, 1896). These often stunningly beautiful fossils commonly retain phosphatised remains of the soft parts. Concerning cephalopods, soft parts have been described from octobrachians and decabrachians (Engeser & Reitner, 1986; Fuchs & Larson, 2011a, 2011b; Fuchs, 2006b; Jattiot et al., 2015; Klug et al., 2021a; Lukeneder & Harzhauser, 2004), ammonites (Hoffmann et al., 2021; Keupp et al., 2016; Wippich & Lehmann, 2004), as well as nautilids (Keupp et al., 2016). Notably, the ectocochleates often display the jaws in situ associated with soft tissue remains. In particular, nautilid and ammonite stomachs with contents have been described repeatedly (Hoffmann et al., 2021; Keupp et al., 2016; Wippich & Lehmann, 2004). Both the lower jaws of ammonites as well as the upper and lower jaws of nautilids bear thickly calcified parts, which are preserved in their original mineralogy (Hoffmann et al., 2021; Keupp et al., 2016; Wippich & Lehmann, 2004). The accompanying soft parts are partially phosphatised, as evidenced by their whitish to bluish fluorescence under UV-light. Some parts also display coatings in rusty brownish to yellowish colours, suggesting the presence of limonite and other iron minerals.

In this paper, we (1) describe two new specimens of the nautilid *Syrionautilus libanoticus* (Foord & Crick, 1890); (2) document their soft tissues; (3) attempt to homologise the visible structures and interpret them, as well as (4) to compare it to modern nautilids.

## Material and methods

C.K. purchased the two specimens from Hussein Ibrahim (Hadjoula) to include them in the collections of the Palaeontological Institute and Museum of the University of Zurich (PIMUZ 37,706 and 37,707). Both specimens consist of part and counterpart, but as so often, most of the material sticks to one of the two sides, which can be advantageous, because different structures may become visible on slab and counterslab. The specimens were photographed by R.R. using both white artificial light and a UVA-handlamp (Hönle UV technology). She used a Nikon D3X with a Nikon AF-S Micro Nikkor 105 mm 1:2.8 objective. R.R. experimented with different distances between the UV-light source and the object. The best results were obtained at distances between UV-light source and object of 40 to 50 cm. She also used UV (UV-Filter MC Lotus from Kaiser Fototechnik) and polarising filters (Nikon Circular Polarizing Filter II), which yielded quite similar results (the purpose is to filter reflected UV-light in order to record only visible light). The UV-photos were automatically corrected for colour in Adobe PhotoShop 2021. Where necessary because the images were too dark, the brightness was increased minimally. More technical details about UV-photography of fossils can be found in Tischlinger and Arratia (2013).

## Results

We describe two specimens of the reasonably common cymatoceratid *Syrionautilus libanoticus* (Foord & Crick, 1890). It shares the strong oblique ribs with the geographically widely distributed Cretaceous nautilid genus *Cymatoceras*, but at least in the Lebanese localities, most specimens of the mentioned species stay small with diameters of 100 mm (Keupp et al., 2016) or less (the specimens described here). Foord and Crick (1890) entitled their original publication “On some new and imperfectly defined species of Jurassic, Cretaceous, and Tertiary nautili […]”, thereby referring to their flattened state and the lack of knowledge of their sutures, which are hardly preserved in platy limestones (Schweigert & Härer, 2021). This preservation causes some uncertainty with respect to the systematic position, but the absence of the shell is fortunate in other respects because it makes jaws and soft-tissue remains visible.

Both specimens presented here preserve jaws (the upper jaw or rhyncholite is visible in both) as well as soft part remains. Although the latter are visible with the bare eye under white light, photography under UV-light revealed important additional details. While PIMUZ 37706 preserves the internal mould of the conch, PIMUZ 37707 shows no trace of it.

**Fig. 1:**
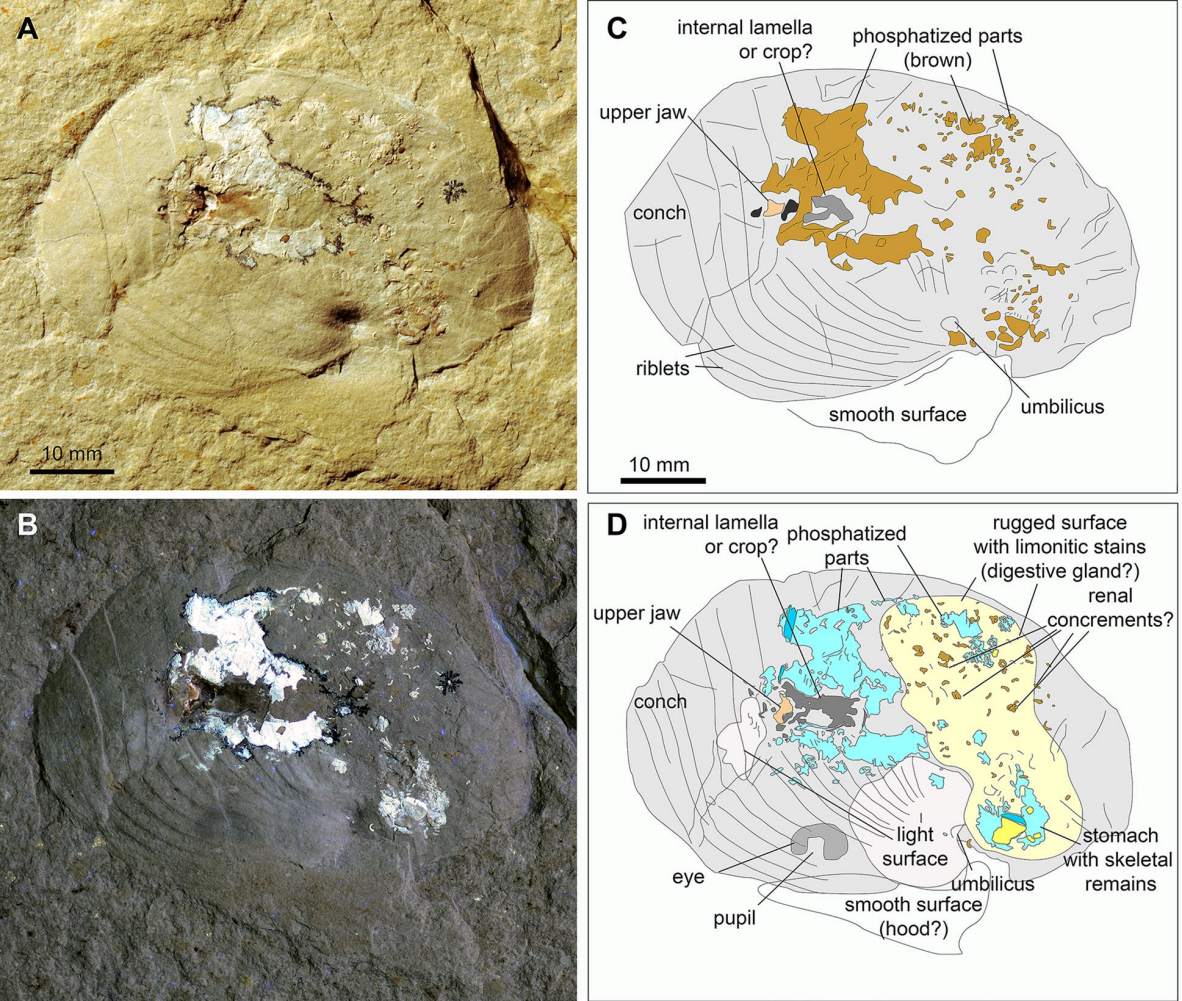
Flattened conch of *Syrionautilus libanoticus* with jaws and phosphatised soft parts in situ; PIMUZ 37706, Cenomanian, Hadjoula, Lebanon. All figures have the same scale. **A** Photo taken under white light; note the ribs and the still discernible umbilicus. **B** Photo taken under UV-light. **C** Line drawing of the structures visible in **A** (light grey—conch imprint without shell material; pink—calcitic part of the upper jaw; dark grey—chitinous wings of the jaws; middle brown—soft part remains with a whitish appearance); the black structures are manganese dendrites and are not drawn. **D** Line drawing of the structures visible in **B** (light grey—structures that appear light greyish under UV-light; middle grey—conch remains; dark grey—the supposed eye and formerly chitinous parts of the jaws that appear dark; turquoise—phosphatised structures with a strong fluorescence under UV-light; light yellow—irregular surface with small fluorescing patches as well as dark patches, possibly limonitic; yellow—possibly calcitic structure)

The conch of PIMUZ 37706 (Fig. 1) has a diameter of 57 mm in its flattened state. The aperture forms a slightly asymmetrical and broadly rounded lateral projection, trailing into a shallow and rounded hyponomic sinus. About 15 ribs are visible, which run roughly parallel to the aperture. They are asymmetrical in cross section with the steeper part facing the aperture. The posterior ribs are visible only adjacent to the nearly punctiform umbilicus (very involute to closed). Due to the deformation, the last whorl is distorted.

The body chamber displays jaw remains as well as phosphatised remains that likely were soft tissues because of their position, shape and mode of preservation (e.g., Keupp et al., 2016; Wippich & Lehmann, 2004). The upper jaw (rhyncholite) is seen from the venter in Fig. 1. Its posterior part is broken. It was 6 mm long and 4 mm wide. The rhyncholite is surrounded by an irregular patch of whitish phosphate, which has a seam of small black manganese oxide dendrites. A brownish tail is visible directly behind the rhyncholite.

Under UV-light, numerous curved and sausage-shaped phosphatised structures are visible, which are irregularly scattered across the posterior body chamber. Between those, small and flat brownish patches are distributed irregularly. Next to the umbilicus, about half a whorl behind the aperture, a second large patch of phosphate is visible, which contains some flat structures as well as additional sausage-shaped structures. Close to the aperture, slightly closer to the umbilicus but still on the midflank, a dark grey oval structure of 7 × 4 mm with a brighter centre is discernible.

**Fig. 2:**
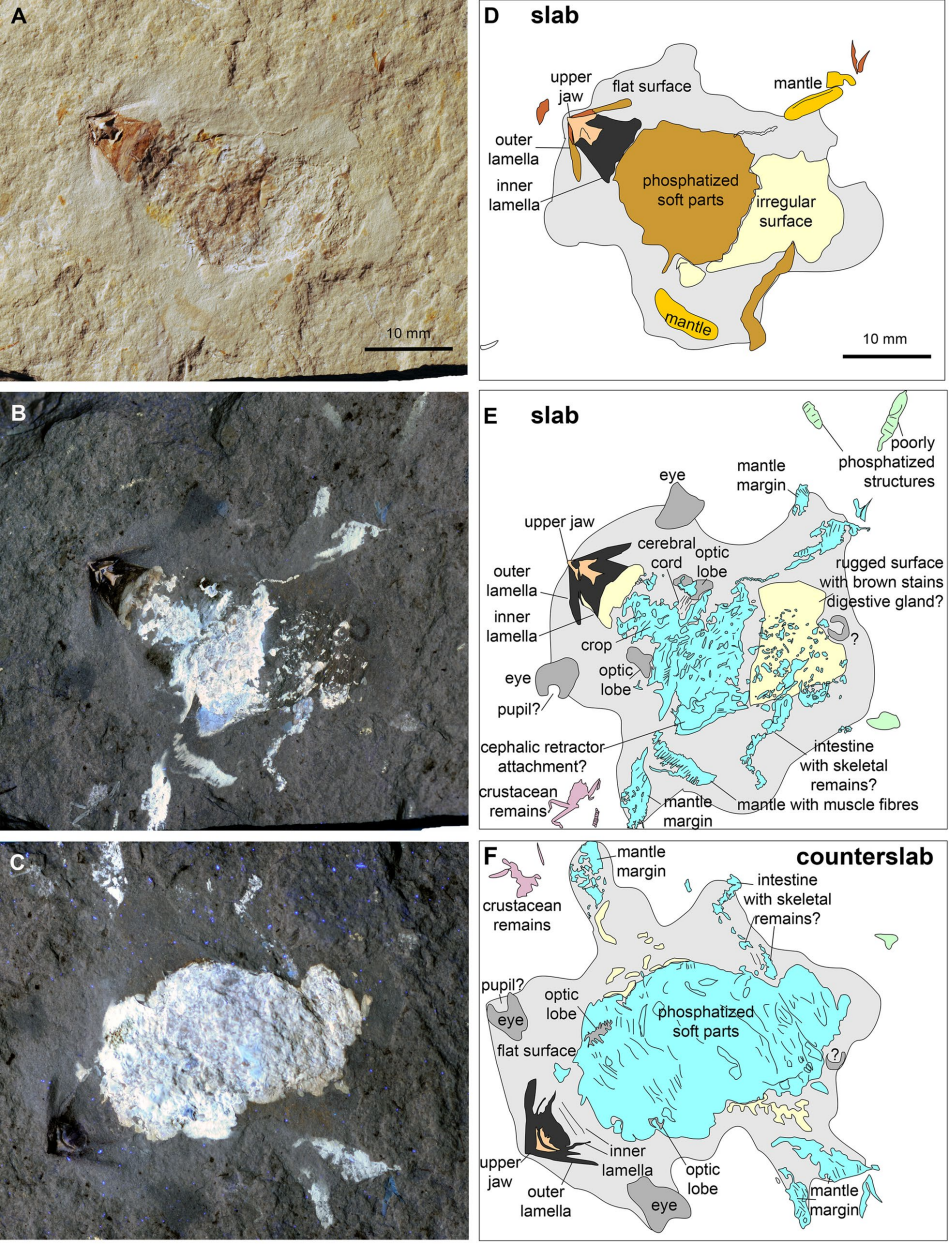
Isolated soft parts of the nautilid *Syrionautilus libanoticus* with the crustacean *Corazzatocarcinus hadjoulae* (mauve in **E**, **F**); PIMUZ 37707, Cenomanian, Hadjoula, Lebanon. All figures have the same scale. **A** Photo of the specimen taken under white light; mainly the rhyncholite is well visible. **B**, **C** Long exposure photo taken with UV light at a distance of 40 cm; note the bright bluish surfaces representing phosphatised structures; the jaw remains appear dark; note the two symmetrically arranged pairs of dark patches next to and behind the jaws. **D** Line drawing of the structures visible in **A** (light grey—even surface surrounding the specimen; pink—calcitic part of the upper jaw; dark grey—inner lamella of upper jaw; middle brown—soft part remains with a brownish appearance; orange—limonite-coloured structures; light yellow—irregular surface with patches of phosphate and possible limonite). **E**, **F** Line drawings of the structures visible in **B** and **C** (light grey—structures that appear middle greyish under UV-light; dark grey—formerly chitinous parts of the jaws that appear dark; turquoise—phosphatised structures with a strong fluorescence under UV-light; light green—phosphatised structures with a weak fluorescence under UV-light; light yellow—irregular surface with small fluorescing patches as well as dark patches, possibly limonitic; light grey—even surface that appears light grey)

The specimen PIMUZ 37707 shows nautilid remains on a surface that is about 41 mm long and maximally 38 mm wide (Fig. 2). Anteriorly, the complete upper jaw is visible. Its remains are distributed over slab (Fig. 2A, B, D, E) and counterslab (Fig. 2C, F) and are described based on the actual specimen and the white light photos, respectively. The rhyncholite (calcitic part) is 4.8 mm long and 5.3 mm wide. The upper jaw preserves the complete inner lamella, which displays a concave striation. In its flattened state, the internal lamella is 9 mm wide and the whole upper jaw is about 9 mm long as well. The outer lamella is preserved only as a symmetrical trace on both sides of the rhyncholite. The furrows have a span of 14 mm.

The soft part remains are visible under white light, but many details are hardly discernible, which become distinct only under UV-light. Thus, these are described based on the photos shown in Fig. 2B, C. Remarkably, the soft part structures are arranged symmetrically, which aided very much in homologisation. The phosphatised soft parts appear whitish to bluish, but there are also symmetrical patches that appear in dark grey in the UV-photo. There is a thickly phosphatised part positioned centrally, which is 29 mm long and 18 mm wide in the counterslab and 18 mm long and 17 mm wide in the slab shown in Fig. 2. In the slab, this part is narrowing anteriorly, where its width is reduced to about 6 mm. Next to this narrow part, a pair of symmetrical dark patches is visible, which are 4 mm long and 2 mm wide each. Between them, a transversely striated structure is preserved, but only faintly phosphatised. Two dark patches are also in a lateral anterior position, roughly at the same level as the upper jaw. These patches are oval and measure about 5 mm in length and width. They share a projection that is directed towards the plain of symmetry and a semicircular to subcircular indentation on the outward oriented side.

Posterior to the smaller dark patches, there is a symmetrical pair of narrow phosphatised lappets, about 1 mm wide and 5 mm long. Behind it, there is a pair of larger, bifurcated lappets at the broadest point of what we interpret as soft tissue remains. The anterior part of these lappets (9 × 3 mm) show a faint transverse striation (i.e. parallel to the former aperture). On the left side (bottom of Fig. 2B), the posterior part of the lappet (9 × 3 mm) shows a distinct longitudinal (i.e. spiral, perpendicular to the sutures and the aperture) striation (25 to 30 short stripes). Also on the left side, a crescent-shaped surface appears in bright blue in Fig. 2B. This surface measures 6 mm in length and about 4 mm in width. On the right side, there is a fine constricted thread, which is 8 mm long and c. 0.5 mm wide. It lies close to the main patch of phosphate and then reaches toward the bifurcated lappet of the right side. Posterior to the strongly phosphatised mass on the slab (Fig. 2B), there is a brownish surface which is 23 mm wide and 17 mm long. It carries many small angular patches of phosphate on the slab and corresponds to a more strongly phosphatised surface in the counterslab (Fig. 2C). On the right side, there is a dark open ring posterior of the other soft parts, which measures 4 mm across and is about 0.7 mm wide. An elongate structure lies opposite of the latter structure. It has an S-form, is about 24 mm long, up to 3 mm wide, and filled with small phosphatic chips. All these structures lie in a flat surface visible under white and UV light, which lacks further mineralised details (light grey in Fig. 2D).

## Discussion

### Taphonomical history

Remarkably, soft part remains are quite commonly preserved in both ectocochleates (ammonites, nautilids) and endocochleates (decabrachian and octobrachian coleoids) in the platy limestones of Hadjoula (Lebanon). These have been described especially for coleoids in great detail (Fuchs & Larson, 2011a, 2011b; Fuchs, 2006a, 2006b; Jattiot et al., 2015; Klug et al., 2020, 2021a; Wippich & Lehmann, 2004). As far as nautilids are concerned, the most detailed account was probably provided by Keupp et al. (2016). These authors provided a photo of a *Syrionautilus libanoticus* with buccal mass and stomach content. In their photo taken under UV-light, no additional details are discernible. Nevertheless, it shows that soft parts are quite often preserved in these ectocochleates.

In most cases, the soft parts are visible inside the conch (Fig. 1), which was dissolved. Like in other platy limestones, the non-preservation of the aragonitic shell enables the examination of internal body parts of ectocochleate cephalopods. Thereby, these fossils reveal anatomical details such as the jaws and sometimes soft part remains. Similar preservation modes of ectocochleates are known from platy limestones of the Carboniferous (Klug et al., 2019; Landman et al., 2010; Mapes et al., 2019), the Late Jurassic (Klug et al., 2016b, 2021a, 2021b; Schweigert, 2009, 2021) and the Late Cretaceous (Ifrim, 2013; Ifrim & Stinnesbeck, 2007; Klug & Lehmann, 2015; Klug et al., 2012, 2020).

Most nautilids and ammonites from the quarry of Hadjoula are preserved similarly to the specimen shown in Fig. 1: the imprint of the flattened conch surrounds the buccal mass, which is usually associated with phosphatised soft tissue-remains, arguably comprising the stomach content (Keupp et al., 2016; Wippich & Lehmann, 2004). This is a common mode of preservation in many platy limestones as stated above.

The soft body was only weakly attached to the inside of the conch, because forward movement of the soft body inside the conch was necessary during growth (e.g., Klug et al., 2008, 2021b; Ward, 1979, 1987). It is thus not surprising that the soft parts fall out of the conch post mortem. According to Ward (personal communication, May 2021), the time between death and the remains falling out depends on water temperature. At 25 °C and above, the remains fall out within about one day. At 16 °C, this process takes longer, based on dead animals in aquaria. However, there is no published record of naturally isolated nautilid soft parts of fossil or recent nautilids to our knowledge. In the fossil record, recognition of isolated soft parts of ectocochleate cephalopods is hampered by the scarcity of soft tissue preservation in general. Hence, usually only the hard parts are recognised (e.g., Sharifi et al., 2021; Tajika et al., 2017; Wilmsen & Yazykova, 2003; Wittler et al., 1999). In the case of isolated jaws, it cannot be ruled out that soft parts were present, which then were either eaten by scavengers or were decaying otherwise (Clements et al., 2016). Also, identifying soft part remains associated with the jaws as such may be difficult depending on the mode of preservation.

As shown recently, soft parts of ectocochleate cephalopods, which became separated from their conch can be fossilised and have a great potential to inform about internal anatomy (Klug et al., 2021b). One of the specimens described here (PIMUZ 37,707; Fig. 2) very likely represents a second case, and the first case of isolated nautilid soft parts. This interpretation is corroborated by the absence of any trace of the conch (which is normally well discernible as seen in Fig. 1) and by the symmetrical arrangement of the soft tissues; in *Syrionautilus*, the soft parts are usually seen either obliquely or laterally on the bedding plane when the conch was present at the time of burial (e.g., Keupp et al., 2016; Wippich & Lehmann, 2004). This is likely linked with the fact that the conchs of both the ammonites and the nautilids from Hadjoula are very narrow with a rounded venter and vertical embedding is accordingly unlikely. Only when embedded fully vertically, such a symmetrical preservation would occur. The finder Hussein Ibrahim (Hadjoula) confirmed that he had never seen this kind of preservation before.

Although we are convinced that the conch was missing at the time of burial, the question remains whether the soft parts were separated from the conch through normal necrolysis or by a predator. The slightly tattered appearance of PIMUZ 37,707 (Fig. 2) and the absence of remains of hood and arm crown somewhat supports the latter interpretation that a predator extracted the soft parts from the conch and dropped parts of it. Accordingly, this would also be a pabulite (leftover fall) sensu Klug et al. (2021b). A second possible interpretation is that a scavenger did not finish its meal for whatever reason (e.g., disturbance by a predator). We can only speculate about the process. Possibly, the predation attempt had a different course than in the case of ammonoids with longidomic conchs such as Jurassic perisphinctids. In the latter case, some predators might have cracked the rear end of the body chamber to facilitate the withdrawal of the soft parts (Klompmaker et al., 2009, 2019). In the case of cephalopods with short body chambers, this mode of predation is unlikely because it would not be very efficient. However, if a predator got hold of the head with the hood and arm crown, some mechanical strain would have been exerted on the soft parts. Alternatively, the arms could have been bitten off or they are not preserved for other reasons. It is conspicuous, however, that the arm crown is not preserved although apparently much of the soft parts is present. It has to be noted here that C.K. saw a nautilid from Hadjoula from a private collection preserving parts of the arm crown. This shows that the potential for arm crown preservation is given at this locality.

### Homologisation of the body parts

Fortunately, the remains of the buccal mass provide evidence for the systematic affinity and the orientation of the organs. In both specimens portrayed here, only the upper jaw is visible. The special preservation conditions made it possible to preserve not only the calcitic rhyncholite, but also the formerly chitinous inner and outer lamellae of the upper jaw (compare Klug, 2001; Saunders et al., 1978).

In the case of PIMUZ 37707 (Fig. 2B, E), the slab displays a longitudinal structure behind the buccal mass, which appears whitish to light bluish under UV-light (Fig. 2B). As already documented in various ammonoid fossils (Klug et al., 2012, 2021b), the crop and oesophagus have a reasonably high fossilisation potential because it is surrounded by chitin. In PIMUZ 37706, a brownish structure is visible in the same position. It is not clear whether this is still the internal lamella or already the crop.

**Fig. 3:**
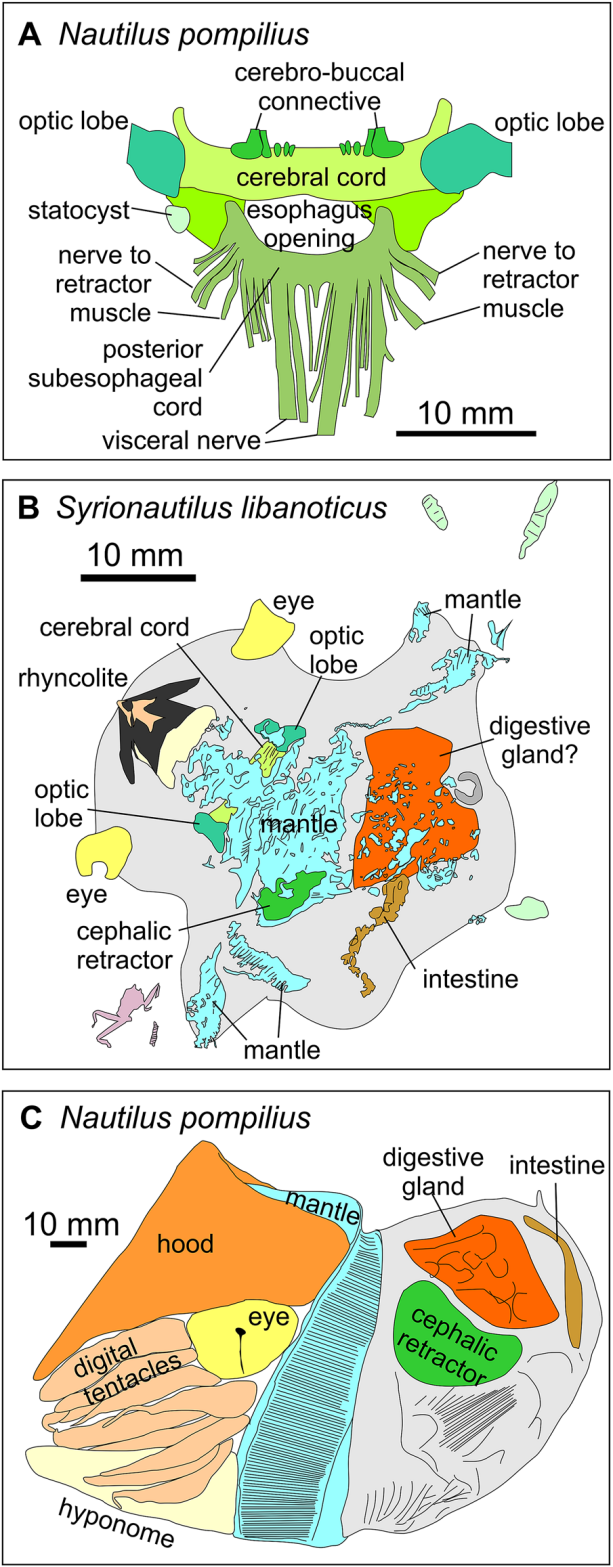
Homologisation of the structures seen in the isolated soft parts of *Syrionautilus libanoticus* with those in *Nautilus pompilius* indicated by the same colours for supposedly homologous structures.** A** Central nervous system of *N. pompilius* drawn after Fig. 14A in Sasaki et al. (2010).** B** Isolated soft parts of *S. libanoticus* from Fig. 1D with different colours. ** C** Lateral view of the isolated soft parts of *N. pompilius* drawn after Fig. 1A in Sasaki et al. (2010)

This anterior part of the digestive tract is surrounded by the brain in cephalopods (Bairati et al., 1987; Nixon & Young, 2003; Sasaki et al., 2010). In PIMUZ 37707, two dark grey spots are arranged symmetrically on both sides of the supposed crop. Their position next to the crop, their shape, the fact that it is a paired structure and their proportions suggest that these are the optic lobes of the brain (compare Sasaki et al., 2010: Fig. 14A). This is further corroborated by a striated structure running perpendicular to the crop, which connects these two rounded structures. This possibly represents the cerebral cord (Fig. 3). In the UV-photo in Fig. 3B, there are two phosphatised structures behind the supposed optic lobes. These could either represent further parts of the central nervous system or parts of the mantle edge. For the pair of dark oval structures and the connection between them, all three homology criteria (relative position, basic structure, same development) are fulfilled. As far as it is visible, this part of the central nervous system looks remarkably similar in size and proportions to that of modern nautilids. This is not so surprising, taking the morphological conservativeness of the Jurassic to modern nautilids (‘living fossils’) into account.

The second pair of structures that appears dark grey in UV-light in Fig. 2B lies on the same level as the buccal mass (yellow in Fig. 3B). Their lateral position at the edge of the soft tissues corresponds well with that of the eyes in modern *Nautilus* (Fig. 3C). Also, their dimensions compared to other body parts support an interpretation as eyes. As far as the homology criterion of specific quality and structure is concerned, the brighter centre on the outward edge suggests that this is either the pupil or it is linked with the fact that the pinhole camera eye is actually filled with sea water, thus there are less tissues in the centre than marginal. The last argument comes from the UV-photo of specimen PIMUZ 37,706, which also displays such a structure, again in a place, where the eyes can be expected (Fig. 1B, D), namely close to the aperture near the formerly broadest part of the conch. Hence, we are quite confident that these structures represent remains of the pinhole camera eyes. Apparently, the morphology of many soft parts such as the central nervous system and the eyes did not change significantly since the Mesozoic.

**Fig. 4:**
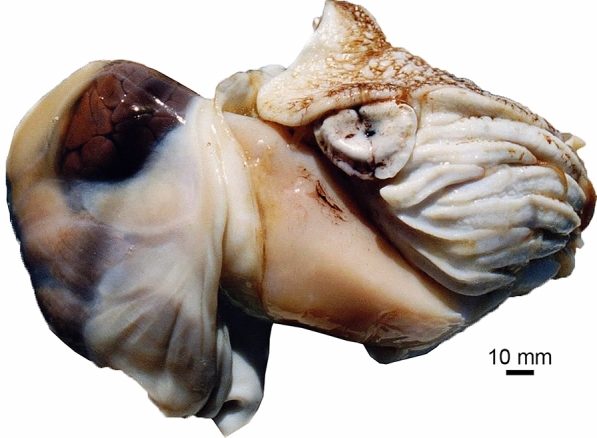
Soft parts of Recent *Nautilus pompilius*, removed from its conch. Note how the mantle extends ventrally in this case. The specimen was kept in deep water for four days in a plastic container to avoid predation. Photo by courtesy of Tomoki Kase (Tokyo)

Both slab and counterslab of PIMUZ 37707 display a prominent feature. These are the aforementioned bilobate lateral lappets. The anterior part is poorly structured with a few longitudinal striae, while the posterior part is finely striated, resembling the pattern seen at the mantle margin in the fossilised mantle of Triassic bivalves (Klug et al., 2005: figs. 10F, 15D). In taphonomic experiments (Wani et al., 2005), isolated soft parts of modern nautilids showed how the mantle becomes loose and a gap to the other soft parts may form (Fig. 4). This may explain the appearance of these mantle remains from the Cenomanian. Accordingly, we see the homology criteria as being fulfilled and suggest that these structures are the mantle margin with the anterior part of the mantle, displaying remains of longitudinal muscle fibres.

Between these mantle remains, there is a mass of phosphate, which displays hardly any discernible structures. Following Keupp et al. (2016), we assume that this mass comprises further parts of the digestive tract (see also Westermann et al., 2002), although we did not see any determinable food remains. In the slabs of PIMUZ 37706 and 37707, there is a surface rich in small thin sheets of brownish iron minerals. Because of their position, we speculate that these might (partially?) represent the digestive glands. There are small phosphatised, bean-shaped structures, aligned in PIMUZ 37707 and randomly distributed over this surface in PIMUZ 37706 (brown in Fig. 1C, D). At first, it was not clear whether these are ichnofossils, small coprolites of a scavenger or cololites of the nautilid itself (cf. Hunt & Lucas, 2012; Hunt et al., 2012; Vallon, 2012; Vallon et al., 2016; Hoffmann et. al. 2020; Knaust, 2020; Knaust & Hoffmann, 2020).

**Fig. 5:**
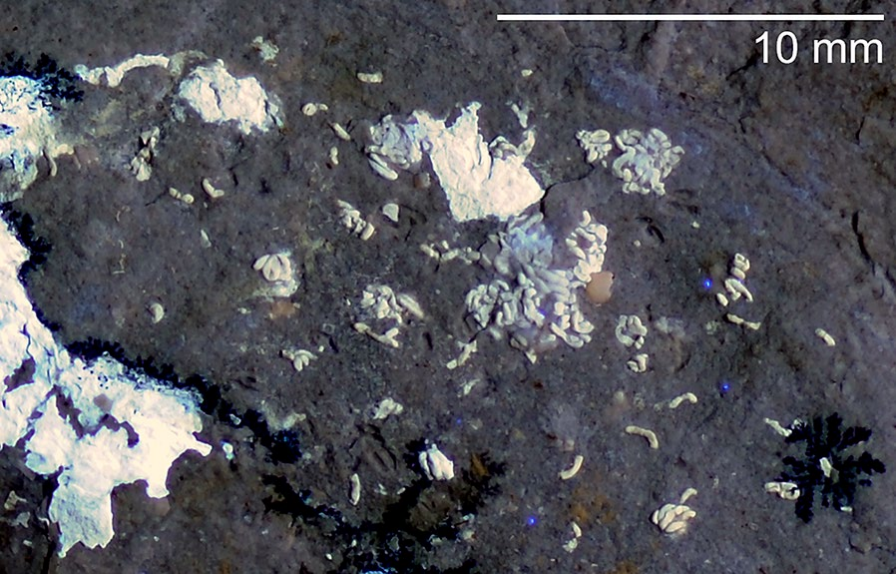
Questionable renal concrements of *Syrionautilus libanoticus*; PIMUZ 37706, Cenomanian, Hadjoula, Lebanon. Detail of Fig. 1B

In PIMUZ 37706 (Figs. 1B and 5), small sausage-shaped phosphatic structures are preserved. These are up to 1 mm long and about 0.2 mm wide. Ward et al (2014) described renal concrements of *Nautilus pompilius*. The concrements depicted by Ward et al., (2014: Fig. 1) are about one order of magnitude smaller, but they are similar in shape, also phosphatic in composition, and they lie in a similar area (center of the body chamber) in the fossil. We suggest that these might be renal concrements as suggested by Ward (personal communication, May 2021), but further research is needed to test this interpretation.

An elongate structure filled with phosphatic chips is preserved in a posterior position in specimen PIMUZ 37707. Because of its position, elongate shape, and the debris filling, we tentatively suggest that this is the intestine. Lastly, there is a circular structure that appears in dark grey under UV-light (marked with a question mark in Fig. 2E).

Finally, there is a faintly brownish structure anterodorsally of the aperture in specimen PIMUZ 37,706 (Figs. 1, 2). We are not sure about its origin, because it lacks any fine structure. Its position, however, allows two alternative interpretations, namely as hood remains or as parts of the phragmocone with the black layer (Klug et al., 2004).

## Conclusions

We describe remains of the nautilid *Syrionautilus libanoticus* from the Cenomanian of Hadjoula, Lebanon. The two specimens are remarkable because they display extensive remains of soft parts. Only one of the two preserves conch remains (mainly the body chamber). The other specimen is stretched out with the plane of symmetry perpendicular to bedding. We interpret this orientation in combination with the incompleteness as another case of a leftover fall (pabulite).

This mode of preservation is so far unique and facilitated the homologisation of some body parts. The homologisation was strongly improved by the use of UV-photos. We suggest that specimen PIMUZ 37707 preserves remains of the buccal mass, much of the digestive tract, the mantle, and, most remarkably, the central nervous system and the eyes. In PIMUZ 37706, small phosphatic structures are tentatively interpreted as renal concrements. These concrements might have served as calcium-source during the formation of new septa.

Our results show the great potential of the Late Cretaceous Konservat-Lagerstätten of Lebanon to preserve anatomical details of soft-tissues unknown otherwise. We are convinced that UV-examination of further ammonites and nautilids from Hadjoula will repeat our findings, help to test our interpretations and will add further important anatomical details. Homologisation of body parts is much easier for fossil nautilids since anatomical differences to modern nautilids are likely minimal compared to, e.g., ammonoids. However, the mode of preservation of organs in nautilids will help to correctly interpret their homologues in the ammonoid fossils of platy limestones in Lebanon and elsewhere.

## Data Availability

Both specimens are stored in the collections of the Palaeontological Institute and Museum of the University of Zurich (PIMUZ numbers).
